# How do Planktonic Particle Collection Methods Affect Bacterial Diversity Estimates and Community Composition in Oligo-, Meso- and Eutrophic Lakes?

**DOI:** 10.3389/fmicb.2020.593589

**Published:** 2020-12-04

**Authors:** Guijuan Xie, Xiangming Tang, Yi Gong, Keqiang Shao, Guang Gao

**Affiliations:** ^1^Taihu Laboratory for Lake Ecosystem Research, State Key Laboratory of Lake Science and Environment, Nanjing Institute of Geography and Limnology, Chinese Academy of Sciences, Nanjing, China; ^2^University of Chinese Academy of Sciences, Beijing, China

**Keywords:** lake, bacterial community structure, bacterial diversity, filtration, centrifugation, particle collection methods, particle-attached bacteria, free-living bacteria

## Abstract

Particles are hotspots of bacterial growth and nutrient recycling in aquatic ecosystems. In the study of particle-attached (PA) and/or free-living (FL) microbial assemblages, the first step is to separate particles from their surrounding water columns. Widely used collection techniques are filtration using different pore size filters, and centrifugation; however, it is unclear how the bacterial diversity, bacterial community structure (BCS) and taxonomic composition of PA assemblages are affected by different particle collection methods. To address this knowledge gap, we collected planktonic particles from eutrophic Lake Taihu, mesotrophic Lake Tianmu, and oligotrophic Lake Fuxian in China, using filtration with five pore size of filters (20, 10, 8.0, 5.0, and 3.0 μm), and centrifugation. Bacterial communities were then analyzed using Illumina MiSeq sequencing of the 16S rRNA gene. We found that PA collection method affected BCS significantly in all lakes. Centrifugation yielded the highest species diversity and lowest mean percentage of photoautotrophic *Cyanobacteria* in Lake Taihu, but not in the other two lakes, thus highlighting the potential compatibility of this method in the study of PA assemblage in eutrophic lakes. The high bacterial diversity and low relative percentage of *Cyanobacteria* was in samples retained on 5.0 μm filters in all lakes. These results suggest that collecting PA samples in lakes using filters with 5.0 μm pore size is the preferred protocol, if species diversity and heterotrophic bacteria are the top research priorities, when comparing bacterial communities in different trophic lakes at the same time. The present study offers the possibility of collecting PA samples using unified methods in oligotrophic to eutrophic lakes.

## Introduction

In aquatic ecosystems, organic particles are pivotal for biogenic element cycling and energy flow (Alldredge et al., 1993; Hodell and Schelske, 1998; Simon et al., 2002; Zhang et al., 2016). Due to the fundamental role in microbial food webs (Azam et al., 1983; Sherr and Sherr, 1988), organic particles have been recognized as the heterogeneous hubs for microbial interactions in oceans and lakes (Bižić-Ionescu et al., 2018). Compared to the surrounding water, the particles are enriched in organic and inorganic matter, and are “hotspots” of microbial growth and remineralization of organic detritus, through the dense colonization and elevated metabolic activities of their attached heterotrophic microbes (Azam et al., 1994; Grossart et al., 2007; Lyons and Dobbs, 2012; Simon et al., 2014). Depending on their relationship with particles, aquatic microbes have traditionally been classified into particle-attached (PA) and free-living (FL) fractions (Mestre et al., 2017). The PA bacterial assemblages are markedly different from their FL counterparts in terms of diversity, structure, taxonomy and function (DeLong et al., 1993; Crump et al., 1999; Allgaier and Grossart, 2006; Smith et al., 2013; D’Ambrosio et al., 2014; Wang et al., 2020).

In the study of PA and/or FL microbial assemblages, the first step is to separate the particles from the surrounding water columns. Therefore, it is vitally important to obtain the most representative PA samples. Large particles (also known as macroaggregates, >500 μm) are usually collected and separated using sediment traps (Thiele et al., 2015) or scuba diving (Smith et al., 1992; Simon et al., 2002). Smaller particles are usually collected and separated by filtration using filters of different pore sizes. At least seven different filter pore sizes have been used to separate PA from FL bacteria: 20 μm (Worm and Søndergaard, 1998), 10 μm (Becquevort et al., 1998; Becquevort and Smith, 2001; Riemann and Winding, 2001; Arnosti et al., 2012), 8.0 μm (Acinas et al., 1997, 1999), 5.0 μm (Selje and Simon, 2003; Allgaier and Grossart, 2006; Parveen et al., 2011; Rösel et al., 2012; Tang et al., 2015; Zhao et al., 2017; Hu et al., 2020), 3.0 μm (Crump et al., 1998, 1999; Herfort et al., 2017; Liu et al., 2019; Liu et al., 2019; Wang et al., 2020), 1.0 μm (Hollibaugh et al., 2000; Zhang et al., 2007), and 0.8 μm (Mevel et al., 2008; Ghiglione et al., 2009). Another method for collecting and separating smaller particles is centrifugation, which has been used in the studied of PA and FL assemblages in turbulent rivers and lakes (Böckelmann et al., 2000; Tang et al., 2009, 2010). Another method in study PA bacterial community is fluorescent *in situ* hybridization. It has been used in freshwater lakes (Lemarchand et al., 2006). Because this method do not need separate particles first, it is not within the scope of current research.

The different particle collection methods and pore size of filters may greatly affect the analyses of microbial communities in aquatic ecosystems. For example, the contradictory results of which habitat (PA vs FL) has the greatest diversity of bacterial species (Acinas et al., 1999; Parveen et al., 2011; Rösel et al., 2012) might be partially explained by use of different methodologies, determining which size range of particles are counted. Moreover, the impacts of particle collection methods on subsequent microbial studies may be trophic-dependent, because nutrients affect the source, composition and size of particles (Tang et al., 2017). Previous studies have revealed that both the volume of water filtered and the different types of filters used have significant effects on DNA extraction efficiency, microbial activity and community structure (Gasol and Moran, 1999; Padilla et al., 2015). However, no systematic analysis has been undertaken of the impacts of different particle collection methods, including pore size of filters, on microbial diversity, community structure, and taxonomic composition.

Therefore, in our present study, we conducted experiments in eutrophic, mesotrophic, and oligotrophic lakes to investigate how bacterial diversity, community structure, and taxonomic composition are affected by particle collection methods. We focused on different filter pore sizes, and on centrifugation. The bacterial communities of eight treatments (six PA fractions and two FL fractions) in each of three lakes were analyzed using Illumina MiSeq sequencing of the 16S rRNA gene. Our findings can be used to minimize the biases associated with particle collection practices in aquatic ecosystems.

## Materials and Methods

### Sample Collection and Environmental Parameters Analyses

**FIGURE 1:**
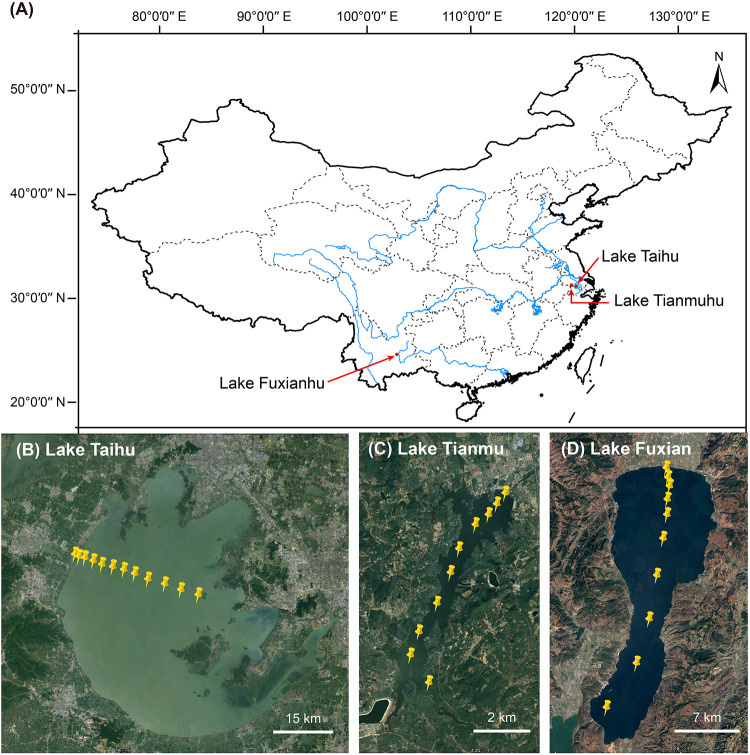
**(A)** Map of China showing the location of the three lakes. Satellite images showing the sampling sites in **(B)** Lake Taihu (*n* = 12), **(C)** Lake Tianmu (*n* = 10), and **(D)** Lake Fuxian (*n* = 10).

In the mid-November 2019, we collected planktonic samples from three lakes: (i) eutrophic, large, shallow, and turbulent Lake Taihu (surface area 2,238 km^2^, mean water depth 1.9 m), (ii) mesotrophic reservoir Lake Tianmu (surface area 12 km^2^, mean water depth 7.0 m), and (iii) oligotrophic deep-water Lake Fuxian (surface area 216 km^2^, mean water depth 89.6 m). The number of sampling sites per lake was 12, 10, and 10 in Lake Taihu, Lake Tianmu, and Lake Fuxian, respectively, (Figure 1). At each site, about 5–20 l of surface water (top 50 cm) were collected. The water samples were transported to laboratory for immediate chemical analysis and subsequent experiments.

At each sampling site, physicochemical parameters of water temperature (WT,°C), electrical conductivity (EC), pH and dissolved oxygen (DO) were measured *in situ* using a multiparameter water quality sonde (YSI EXO2, Yellow Springs Instruments Inc., United States) at approximately 50 cm depth. Transparency (SD) were measured using the Secchi disk. Total nitrogen (TN), total dissolved nitrogen (TDN), total phosphorus (TP), total dissolved phosphorus (TDP), chlorophyll*-a* (Chl-*a*), chemical oxygen demand (COD_*Mn*_, permanganate index), dissolved organic carbon (DOC), total suspended solids (TSS), and loss of ignition (LOI), were measured according to standard methods (Jin and Tu, 1990; Baird et al., 2017). The relative percentage of organic matter (OM) in TSS was calculated using the following formula: (TSS – LOI)/TSS × 100%.

### Enumeration of Bacteria and Particles

Raw lake water and the 5.0 μm pre-filtered filtrate were fixed with formaldehyde (a final concentration of 2%) and used to enumerate the abundances of total bacteria (TB) and free-living bacteria (FLB), respectively. TB and FLB were enumerated by a flow cytometer according to Gong et al., 2017. Then, the abundance of particle-attached bacteria (PAB) was calculated as TB minus FLB. For the abundance of detrital particles (Tang et al., 2009), the fixed raw water samples were stained by 4′,6′-diamidino-2-phenylindole (DAPI, Sigma) at a final concentration of 2 μg/ml for 10 min, filtered onto black polycarbonate filters (0.2-μm pore size, 25-mm diameter, Poretics)^TM^ and enumerated using Axio Imager A2 epifluorescence microscope (Zeiss, Germany) equipped with a high speed, high resolution and sensitivity camera (Hamamatsu, Janpan). At least 100 particles (diameter > 5 μm) were counted and be photographed. The acquired particle images were analyzed by HCimage live software. Then the abundance of particles, mean diameter and mean area of particles in each lake were calculated.

### Experimental Design

**FIGURE 2:**
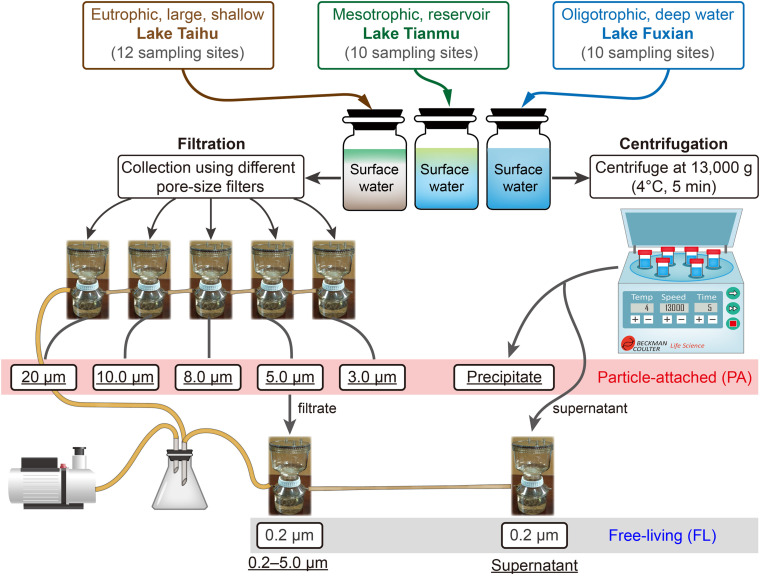
The schematic diagram of experiment design, and the different treatments for collecting particle-attached (PA, six treatments in the red box) from Lake Taihu, Lake Tianmu, and Lake Fuxian. The <5.0 μm-filtrate and the supernatant after centrifugation are subsequently filtrated by 0.2 μm filter membranes and are considered as free-living (FL, in the gray box) bacterial samples. The terms underlined represent the designated names of the treatments.

For each lake, we mixed all the water samples, using an equal volume from each site. Then, eight protocols were used for each lake: six for PA samples (five filtration and one centrifugation), and two for FL samples (one filtration and one centrifugation) as controls of PA samples (Figure 2). For each protocol, there were four replicates. Thus 96 samples were anticipated: 3 lakes × 8 protocols × 4 replicates. Our final total number of samples was 94, because for lake Fuxian, we combined four replicates into two samples for DNA extraction and sequencing, due to relatively low precipitates when using centrifugation.

For filtration of PA bacteria, we used five different pore size filters, i.e., 20 μm nylon net, 10, 8.0, 5.0, and 3.0 μm polycarbonate membranes (all 47 mm in diameter, Merck Millipore Ltd., Supplementary Table 1). For centrifugation of PA bacteria, we centrifuged at 13,000 *g* at 4°C for 5 min (Böckelmann et al., 2000). The FL bacteria were collected from the 5.0 μm pre-filtered filtrate and the supernatant after the centrifugation (Figure 2), by filtering through 0.2 μm polycarbonate membrane (Merck Millipore Ltd.).

Due to different concentrations of particles in the three lakes (Table 1), the water volume used and filtration processes were different. For Lake Taihu, 300 ml of mixed water were used for each of the five filtrations, and for the centrifugation. A single filter for the 20 μm sample. Two filters were used for collecting the 10, 8.0, 5.0, and 3.0 μm samples to avoid clogging. For Lake Tianmu, 300 ml of mixed water were used for each of the five filtrations (a single filter for each sample), and for the centrifugation. For Lake Fuxian, 1,500 ml of mixed water were used for each of the five filtrations (a single filter for each sample), and for the centrifugation. All filtrations were performed using a Nalgene^®^ filter holder with 500 ml receiver, connected to an oil-free vacuum pump (ChemVak V400, WIGGENS, Germany) under a vacuum < 200 mmHg. All filters and precipitates contained bacterial communities were stored at −80°C in the laboratory until DNA extraction.

**TABLE 1:** Mean values (mean ± SD) and comparisons among lakes (Kruskal–Wallis test) of main environmental parameters, bacterial abundance, and characteristics of particles in November 2019.

	**Lake Taihu**	**Lake Tianmu**	**Lake Fuxian**
Main environmental parameters			
TN (mg/l)	1.880.91^a^	0.650.04^b^	0.160.01^c^
TP (mg/l)	0.1410.064^a^	0.0320.008^b^	0.0090.001^c^
Chl-*a* (μg/l)	23.415.2^a^	22.36.9^a^	5.30.7^b^
TSS (mg/l)	51.537.4^a^	9.55.6^b^	0.70.1^c^
OM (%)	18.911.8^a^	39.212.4^b^	85.76.7^c^
Bacterial abundance			
TB (10^6^ cells/ml)	12.703.42^a^	10.012.45^b^	2.830.16^c^
PAB (10^6^ cells/ml)	6.041.78^a^	3.470.55^b^	0.640.08^c^
Percentage of PAB (%)	47.43.7^a^	35.55.3^b^	22.83.3^c^
Characteristics of particles			
Particle abundance (10^4^ individuals/ml)	27.114.2^a^	6.61.3^b^	1.10.3^c^
PAB per particle (individuals)	2916^a^	539^b^	6112^b^
Mean diameter (μm)	165^a^	193^ab^	269^b^
Mean area (μm^2^)	7448^a^	8625^ab^	118102^b^

*TN, total nitrogen; TP, total phosphorus; Chl-*a*, chlorophyll-*a*; TSS, total suspended solids; OM, organic matter; TB, total bacteria; and PAB, particle-attached bacteria. After each number, the different lower-case letters indicate significant differences (*P* < 0.05) among different lakes.*

### Molecular Analyses

We extracted DNA using FastDNA^®^ Spin Kit for Soil (MP Biomedicals, United States) according to the manufacturer’s instructions. The V3-V4 hypervariable regions of the bacterial 16S rRNA genes were amplified using the primer set 338F (5′-ACTCCTACGGGAGGCAGCAG-3′) and 806R (5′-GGACTACHVGGGTWTCTAAT-3′; Fadrosh et al., 2014). The polymerase chain reaction (PCR) mixtures contained 20 ng diluted DNA template, 0.4 μM of each primer, and NEB phusion high-fidelity PCR master mix. The PCR program was as follows: 3 min of initial denaturation at 98°C followed by 30 cycles of denaturation at 98°C (45 s), annealing at 55°C (45 s), elongation at 72°C (45 s), and a final extension at 72°C for 7 min. Following purification of the amplicon pools using AMPure XP beads, sequencing was consequently performed on the Illumina MiSeq PE300 platform by the Beijing Genomics Institute (BGI), China, using a pair-end sequencing (2 × 300) strategy.

### Bioinformatics and Statistical Analysis

The bioinformatic analysis was carried out on CLC Genomics Workbench 20.0 (Qiagen) following standard workflows of “OTU Clustering Using Workflows^1^”. After importing raw reads, a standard analysis including the following four steps, trim, merge paired reads, trim to fixed length, and filter samples based on number of reads, were carried out step by step (Christensen, 2018). Then, bacterial phylotypes were identified and assigned to operational taxonomic units (OTUs, 97% similarity). To minimize the random sequencing error, we filtered out low abundance (<10 reads) OTUs from the OTU table. Taxonomy classification was performed by comparing reads against the SILVA small subunit rRNA (SSU) database v132 at 80% confidence level (Quast et al., 2013). Sequences associated with chloroplast were excluded from subsequent analysis.

Bacterial α-diversity (Chao1 richness and Shannon index) were calculated at a uniform sequence depth. To evaluate the bacterial community structure (BCS) among different treatments, non-metric multidimensional scaling (nMDS) analyses and cluster analyses of Bray–Curtis distance were performed using the PRIMER-E (v7) software package (Clarke and Gorley, 2015).

The statistical analyses were performed using R 3.5.3^2^ with RStudio 1.1.463 interface, unless otherwise indicated. Data visualization was performed using the packages “*ggplot2*” in R. To examine the differences of physicochemical parameters among the three lakes, the differences of bacterial α-diversity, and percentages of main bacterial phyla (calculated based on the number of OTU reads affiliated to the phyla) among treatments, we performed the non-parametric Kruskal–Wallis test with Holm correction of *P*-values for multiple comparisons. To test the difference of BCSs among treatments, based on the bacterial Bray-Curtis similarity distance matrix, we performed analysis of similarity (ANOSIM) with 9,999 permutations (Clarke, 1993). The ANOSIM then generates a test statistic *R*, with a score of 1 indicating complete separation, and 0 indicating no separation. A *P*-value less than 0.05 is reported as statistically significant.

### Data Deposition

Raw sequence data reported in this paper have been deposited in the Genome Sequence Archive in the BIG Data Center (National Genomics Data Center Members, and Partners, 2020) under accession number CRA003056, and publicly accessible at http://bigd.big.ac.cn/gsa.

## Results

### Environmental Characterization

Main environmental parameters, bacterial abundance and characteristics of particles are presented in Table 1. There was a distinct nutrient (TN and TP) gradient among the three lakes. The concentrations of Chl-*a* in Lake Taihu and Lake Tianmu were about 4 times of that in Lake Fuxian. In addition, the concentrations of TSS in Lake Taihu was about 5 and 74 times of those in Lake Tianmu and Lake Fuxian, respectively. However, the relative percentage of OM in TSS in Lake Fuxian was about 4.5 times than that in Lake Taihu. Other nine physicochemical parameters for the three lakes are presented in Supplementary Figure 1.

The mean abundances of PAB in Lake Taihu, Lake Tianmu and Lake Fuxian were 6.04, 3.47, and 0.64 × 10^6^ cells/ml, while the mean abundances of particles in the three lakes were 27.1, 6.6, and 1.1 × 10^4^ individuals/ml, respectively. The percentage of PAB decreased from eutrophic Lake Taihu to oligo-trophic Lake Fuxian, while PAB per particle, mean diameter and mean area of particles had the opposite trend, i.e., increased with the decreasing trophic levels.

### Bacterial α-Diversity Among Different Treatments

**FIGURE 3:**
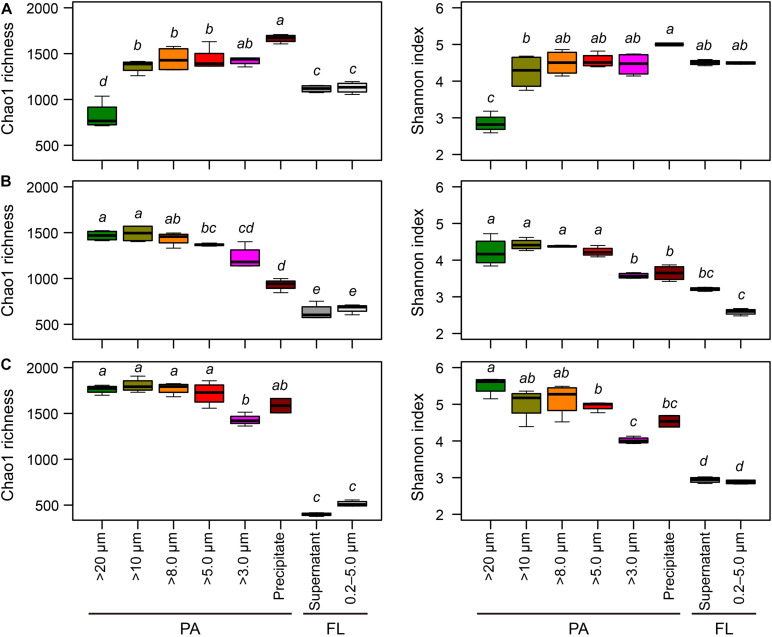
Boxplots of the Chao1 richness and Shannon diversity indices for each sample collection treatment in **(A)** Lake Taihu, **(B)** Lake Tianmu, and **(C)** Lake Fuxian. Diversity indices were calculated using subset of 19,687 sequences per sample. The median values are shown with horizontal black wide lines. Upper and lower edges of the boxes represent the first and third quartile of the distribution of values, with whiskers indicating the lowest and highest values within 1.5 times the interquartile range of the lower and quartiles, respectively. Kruskal–Wallis test was performed to examine the differences among treatments with Holm correction of *P*-values for multiple comparisons. Different lower-case letters above each boxplot indicate significant differences (*P* < 0.05) among treatments. PA, particle-attached; FL, free-living.

After quality control, we generated 2,896,775 high quality reads, with a mean of 30,817 reads per sample (range 19,687 to 43,406). The reads were classified into 3,717 OTUs across the 94 samples. The Chao1 richness and Shannon diversity indices varied significantly with different sample treatments in the three lakes (Figure 3). In all lakes, bacterial richness was significantly lower in the FL habitat than in the PA habitat (Kruskal–Wallis test, *P* < 0.001). However, among the three lakes, within the PA habitat, different particle collection methods had different impacts on estimates of Chao1 richness and Shannon diversity.

In Lake Taihu, centrifugation yielded the highest richness of PA bacteria, while the samples retained on 20 μm filters had the lowest richness (Figure 3A). In contrast, in Lake Tianmu centrifugation yielded the lowest richness of PA bacteria, while samples retained on 20, 10, and 8.0 μm filters has the highest richness (Figure 3B). In Lake Fuxian, the samples retained on 3.0 μm filters had the lowest richness of PA bacteria; there were no significant differences in Chao1 richness among PA samples processed with other methods (Figure 3C).

For Shannon diversity, in Lake Taihu, the samples retained on 20 μm filters had the lowest diversity. In contrast, in both Lake Tianmu and Lake Fuxian, the samples retained on 3.0 μm filters had the lowest diversity.

### Bacterial Community Structure Among Different Treatments

**FIGURE 4:**
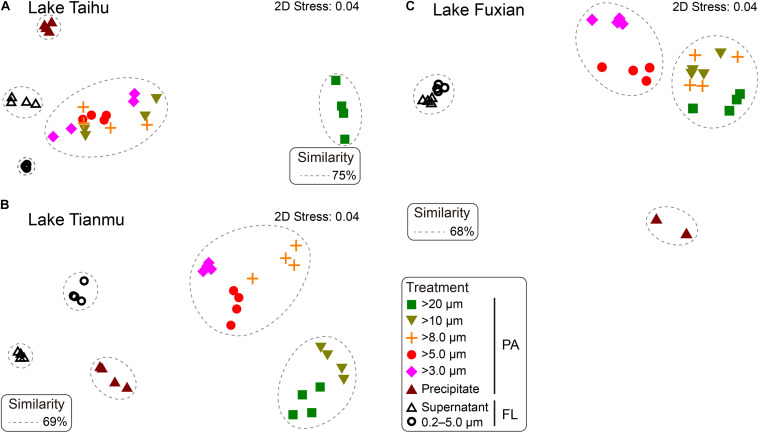
Non-metric multidimensional scaling (nMDS) showing the distance between samples generated from different treatments in **(A)** Lake Taihu, **(B)** Lake Tianmu, and **(C)** Lake Fuxian. Samples with Bray-Curtis similarities of 75% in Lake Taihu, 69% in Lake Tianmu, and 68% in Lake Fuxian, respectively, are circled with dashed lines. PA, particle-attached; FL, free-living.

In all three lakes, the BCS varied significantly with habitat (PA vs FL) and sample collection method (Figure 4 and Supplementary Table 1). The ANOSIM showed that the differences of BCSs between PA and FL samples were statistically significant (*P* < 0.05). The BCSs of PA samples collected using centrifugation were significantly different (*P* < 0.05) from those collected using filtration. Furthermore, there were differences between lakes in the variations among PA samples collected using filters with different pore sizes.

In Lake Taihu, the BCS of samples retained on 20 μm filters was significantly different from the other filtered samples (Figure 4A). The samples retained on the 10, 8.0, 5.0, and 3.0 μm filters shared their bacterial communities with similarity of 75%; ANOSIM did not reveal any significant differences among these four treatments (*P* > 0.05, Supplementary Table 1). In Lake Tianmu, samples retained on 20 μm and 10 μm filters clustered together, with a similarity of 69%, while samples retained on 8.0, 5.0, and 3.0 μm filters formed another cluster (Figure 4B); ANOSIM revealed that the differences among all treatments were statistically significant (*P* < 0.05). In Lake Fuxian, samples retained on 20, 10, and 8.0 μm filters formed one cluster, with a similarity of 68%, while samples retained on 5.0 μm and 3.0 μm filters formed the second cluster; samples from the two FL treatments formed the third cluster (Figure 4C). The ANOSIM revealed statistically significant differences among all treatments (*P* < 0.05), except 10 μm and 8.0 μm pore size filters.

### Bacterial Taxonomy Among Different Treatments

**FIGURE 5:**
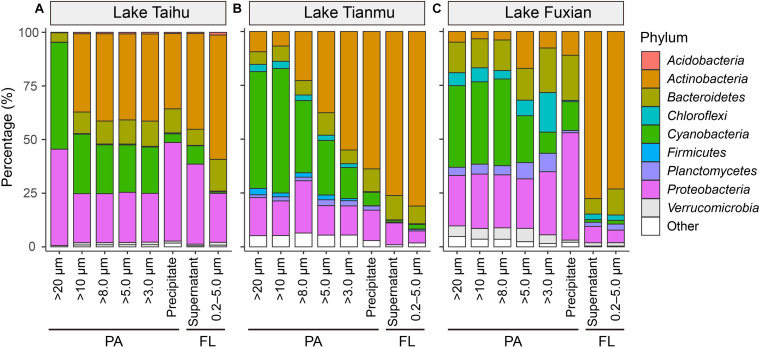
Mean percentage (%) of major bacterial taxa in each treatment at phylum level in **(A)** Lake Taihu, **(B)** Lake Tianmu, and **(C)** Lake Fuxian. The nine most abundant bacterial phyla are shown individually, and minor taxa are combined as “other”. PA, particle-attached; FL, free-living.

In all three lakes, there were significant differences in the percentages of major bacterial taxa with habitat (PA vs FL), and with sample collection methods (Figures 4, 5). The relative abundance of *Actinobacteria* was significantly higher in the FL habitat than in the PA habitat, while *Cyanobacteria* and *Proteobacteria* were significantly more abundant in the PA than FL habitat.

**FIGURE 6:**
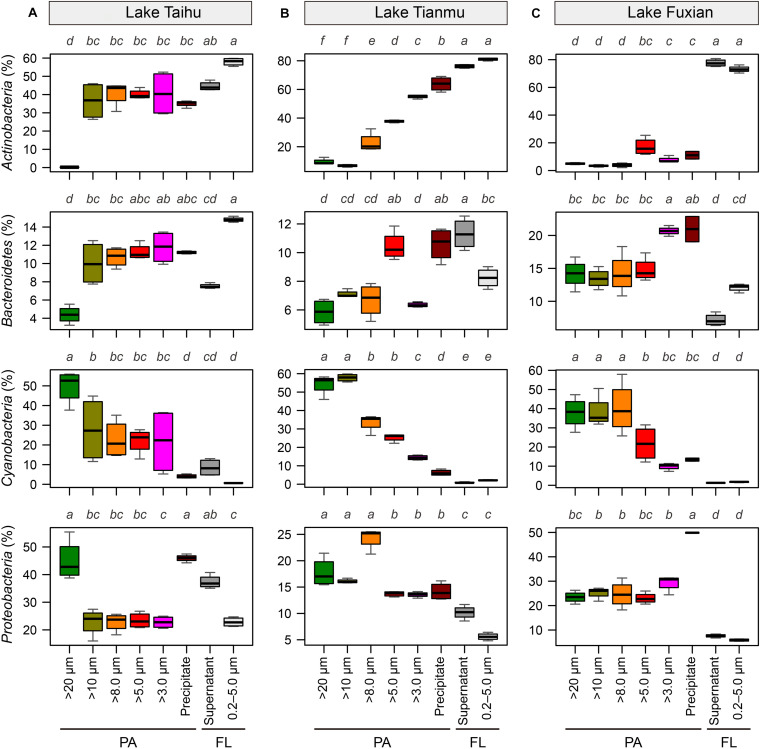
Boxplots of the major four bacterial phyla for each sample collection treatment in **(A)** Lake Taihu, **(B)** Lake Tianmu, and **(C)** Lake Fuxian. Note variation in *y*-axis scales in different lakes. To examine the differences between treatments, the Kruskal–Wallis test was performed with Holm correction of *P*-values for multiple comparisons. Different lower-case letters above each boxplot indicate significant differences (*P* < 0.05) among treatments. PA, particle-attached; FL, free-living.

In all three lakes, for the PA habitat, the different particle collection methods had different impacts on the percentages of major bacterial taxa. In Lake Taihu (Figure 6A), the percentages of *Actinobacteria* and *Bacteroidetes* in the samples retained on 20 μm filters were significantly lower compared with other treatments (*P* < 0.01), while the percentage of *Cyanobacteria* was significantly higher (*P* < 0.05). The percentages of *Proteobacteria* in the samples retained on 20 μm filters and collected by centrifugation were approximately double that in other PA samples collected using filtration with pore sizes of 10, 8.0, 5.0, and 3.0 μm.

In Lake Tianmu (Figure 6B), centrifugation yielded significantly higher percentage of *Actinobacteria* and lower percentage of *Cyanobacteria* than did filtration. Among the samples collected using filtration, the percentage of *Cyanobacteria* was significantly higher in samples retained on 20 μm and 10 μm filters than on other pore size filters, while the percentage of *Proteobacteria* was significantly lower in samples retained on 5.0 μm and 3.0 μm filters than on 20, 10, and 8.0 μm filters.

In Lake Fuxian (Figure 6C), significantly higher percentage of *Actinobacteria* and lower percentage of *Cyanobacteria* were recorded in samples retained on 5.0 μm and 3.0 μm filters, and by centrifugation, than those retained on 20, 10, and 8.0 μm filters. The centrifugation method also yielded a significantly higher percentage of *Proteobacteria* than did filtration.

**FIGURE 7:**
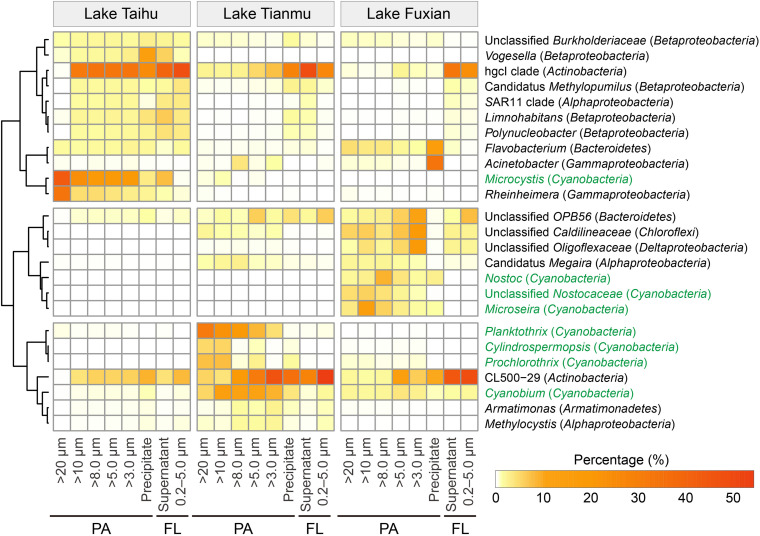
Heatmap of the 25 major genera, including the top 10 genera in each lake. Genera in green are associated with the photosynthetic autotrophic *Cyanobacteria*. PA, particle-attached; FL, free-living.

Heatmap of the 25 major genera (including the top 10 genera in each lake) showed that the percentages of heterotrophic and autotrophic (within the *Cyanobacteria* phylum) bacterial genera were distinctly different among treatments, as well as among different trophic lakes (Figure 7).

## Discussion

Our experimental results demonstrate that particle collection methods significantly affect bacterial diversity estimate, BCS, and taxonomic compositions in lake ecosystems. Furthermore, the extent of impacts was related to the trophic level of the lake.

### Centrifugation Is Suitable for Collecting Particles in Eutrophic and Turbulent Aquatic Ecosystem

One important finding is that centrifugation was more suitable than filtration for collecting particle samples in a eutrophic and turbulent aquatic ecosystem with high concentration of TSS, as exemplified by Lake Taihu. We suggest three advantages for this. (1) Samples collected by centrifugation (Precipitate, Figure 2) had the highest diversity in eutrophic Lake Taihu, with a mean TSS concentration of 51.5 mg/L (Figure 3A and Table 1). (2) The percentage of *Cyanobacteria* from centrifugation was significantly lower than that from filtration treatments (Figures 5, 6), which would benefit observations focused on heterotrophic bacterial assemblages. In Lake Taihu, the dominant cyanobacterial genus was *Microcystis* (Figure 7) which can form large buoyant colonies. Due to the high buoyancy, *Microcystis* colonies generally float on the water surface after centrifugation. Large *Microcystis* colonies (>20 μm) can reach up to 96% of the total cyanobacterial cells in this lake (Wu and Kong, 2009; Qin et al., 2016), which could explain the high proportion of *Cyanobacteria* (mean = 50%) in Lake Taihu samples retained on 20 μm filters (Figure 5A). Moreover, we found significantly lower diversity (Figure 3A) and significantly different BCS (Figure 4A) in samples detained on 20 μm filters compared with other treatments, which was consistent with other studies on *Microcystis* colony-associated bacteria (Shi et al., 2012; Parveen et al., 2013; Shi et al., 2018). The increased species diversity associated with decreasing pore size of filters (from 20 μm to 3.0 μm, Figure 3A) confirmed that most heterotrophic bacteria in Lake Taihu were attached to small particles (Tang et al., 2009). (3) Diversity in the supernatant (Figure 2) after centrifugation was not significantly different than the FL samples (0.2–5.0 μm) collected using filtration (Figure 3), indicating that centrifugation could effectively separate PA bacteria from FL assemblage in Lake Taihu.

Previous studies have also demonstrated that centrifugation can capture bacterial assemblages attached to particles, such as the reports for the Elbe river in Germany (Böckelmann et al., 2000), an acidic mine lake in Germany (Reiche et al., 2011), and Lake Taihu in China (Tang et al., 2009, 2010). However, a note of caution is due here because in our present study, the BCS of samples collected using centrifugation (Precipitate) were significantly different from the PA samples collected using filtration methods in all three studied lakes (Figure 4). Centrifugation could enrich some bacterial taxa disproportionately, probably because of their shape and/or size, which is related to sinking velocity under centrifugation. For example, the percentage of bacteria associated with the genus *Vogesella* (*Betaproteobacteria*) was significantly enriched (Kruskal–Wallis test, *P* < 0.05) in samples collected using centrifugation in Lake Taihu (Figure 7). Members of the genus are rod-shaped, gram-negative bacteria with the average cell dimensions of 0.5 by 3.5 μm (Grimes et al., 1997). *Vogesella* strains are capable of degrading peptidoglycan, a few monosaccharides and other polymer compounds (Grimes et al., 1997; Jørgensen et al., 2010), which are typical secretions of cyanobacterial colonies. Using the Advanced BLAST search program on the National Center for Biotechnology Information (NCBI) website, we found that the most abundant OTU in Precipitate samples in Lake Taihu (accounting for 13% and 98% of the total and the *Vogesella* genus sequences, respectively) shared 100% sequence similarity with the strain of *Vogesella* sp. AKB-2008-TE15 (NCBI accession No. AM989119), previously isolated from lake water with cyanobacterial blooms (Berg et al., 2009).

Another important finding is that the diversity indices of samples collected using centrifugation and filtered through 3.0 μm filters were significantly lower than those retained on 20 to 5.0 μm filters in mesotrophic Lake Tianmu and oligotrophic Lake Fuxian (Figure 3). These results suggest that the centrifugation and the filtration through 3.0 μm filters were not suitable for collecting PA bacteria in mesotrophic and oligotrophic lakes if species diversity is the top priority.

The low diversity in samples collected using centrifugation in mesotrophic and oligotrophic lakes may be driven by several mechanisms related to the characteristics of particles. (1) Due to low nutrient availability, the phytoplankton community, concentration of TSS and percentage of organic matters in particles in mesotrophic and oligotrophic lakes differed from that in eutrophic lakes. In our present study, *Planktothrix*, *Cylindrospermopsis*, and *Prochlorothrix* were the dominant cyanobacterial genera in mesotrophic Lake Tianmu, while *Nostoc*, *Microseira*, and *Cyanobium* were dominant in oligotrophic Lake Fuxian, and *Microcystis* was dominant in eutrophic Lake Taihu (Figure 7). In addition, the concentrations of TSS in Lake Fuxian and Lake Tianmu were significantly lower than that in Lake Taihu (Table 1). Within the TSS, however, the mean percentage of organic matter in Lake Fuxian and Lake Tianmu was 86% and 39%, respectively, (Table 1), which is significantly higher than that in Lake Taihu (19%). The high percentage of organic matter in the TSS may provide more resistance to sinking down during centrifugation compared with TSS with abundant inorganic matter. (2) We found the highest Chao1 richness of samples retained on 10 μm filters in both Lake Tianmu and Lake Fuxian (Figures 3B,C), indicating that heterotrophic bacteria were more densely colonized on large organic particles in these two lakes than that in Lake Taihu (Table 1). This finding is consistent with previous studies showing that particles in oligotrophic marine and deep lakes are generally rich in organic detrital matter which is densely colonized by bacteria, while particles in turbulent estuaries or large shallow lakes tend to be smaller with significant content of inorganic matter (Simon et al., 2002; Zhang et al., 2016).

Surprisingly, the diversity indices of the PA samples retained on 3.0 μm filters in both Lake Tianmu and Lake Fuxian were significantly lower than those retained on large pore size filters (Figure 3), because smaller pore size filters are expected to retain more size ranges of bacteria. The porosity of 3.0 μm filters is 11.3%, which is different than that of the 10, 8.0, and 5.0 μm filters (5–20%; Supplementary Table 2). Different porosity may be one of the reasons that resulted in low diversity of PA samples detained on 3.0 μm filters. We noticed a faster filtration rate when using 3.0 μm filters than when using 8.0 μm and 5.0 μm filters in Lake Tianmu and Lake Fuxian. Our results suggest that collecting particles using 3.0 μm filters in mesotrophic and oligotrophic lakes is not suitable if species diversity is the top priority, though the potential mechanisms for this difference need to be explored further.

### Collecting Particles Using 5.0 μm Filters Is the Optimal Choice When PA Bacterial Communities in Different Trophic Waterbodies Are Compared at the Same Time

Even though previous paragraphs of the discussion suggested that the PA collection method will depend on the outcome of interest, and on the trophic status of the water body that is being sampled, the present study raises the possibility of collect PA samples using a unified method. If the bacterial communities in lakes of different trophic status are compared at the same time, our results suggest that collect PA samples using 5.0 μm filters is the optimal choice, considering the comprehensive factors of diversity, taxonomic composition, and the discrimination between PA and FL assemblages. There are several reasons supported this recommendation. (1) Bacterial diversity indices of samples retained on 5.0 μm filters were consistently among the highest compared to those derived using other pore size filters (Figure 3). (2) The relative abundance of *Cyanobacteria* in samples retained on 5.0 μm filters was significantly lower than those using: 20 μm filters in Lake Taihu; 20 μm and 10 μm filters in Lake Tianmu; and 20, 10, and 8.0 μm filters in Lake Fuxian (Figures 5, 6). Thus, use of 5.0 μm filters would benefit the studies that focus on heterotrophic bacterial assemblages. (3) The BCSs of PA samples retained on 5.0 μm filters were significantly different from those of FL samples (0.2–5.0 μm) in all three lakes (Figures 4, 7), indicating a distinct discrimination of PA and FL bacterial communities using 5.0 μm filters.

In this study, the well-known methodological constraints on separation of PA and FL assemblages must be considered (Tang et al., 2015; Mestre et al., 2017). During sampling using filtration or centrifugation, the FL bacteria could be detained on filters because of clogging, meanwhile PA bacteria could enter into the FL fraction due to detachment from particles (Padilla et al., 2015). Both processes would increase the similarities between PA and FL bacterial communities. Moreover, many aquatic bacteria may possess an alternate lifestyle between a PA and FL stage (Grossart, 2010). Despite the uncertainty of methodology, we argue that the observed patterns of bacterial diversity and community composition among different particle collection methods are conserved, and could be a null hypothesis to be tested in other different trophic lakes. Based on our findings, we encourage researchers to collect PA samples using 5.0 μm filters for the comparisons of the diversity, function and underlying regulation mechanisms of PA and FL bacteria in aquatic ecosystems with different trophic levels.

## Data Availability Statement

Raw sequence data generated by this study can be found in the BIG Data Center (https://bigd.big.ac.cn/gsa/browse/CRA003056).

## Author Contributions

XT and GG designed the study. GX, KS, and XT collected the samples. GX, YG, KS, and XT analyzed the data. GX and XT wrote the manuscript, with contributions from all co-authors. All authors have approved the final manuscript for publication.

## Conflict of Interest

The authors declare that the research was conducted in the absence of any commercial or financial relationships that could be construed as a potential conflict of interest.

## Footnotes

1 https://resources.qiagenbioinformatics.com/tutorials/OTU_Clustering_Workflows.pdf

2 https://www.r-project.org
