# Supplementary material for: How do Planktonic Particle Collection Methods Affect Bacterial Diversity Estimates and Community Composition in Oligo-, Meso- and Eutrophic Lakes?
Source: Front Microbiol. 2020 Dec 4;11:593589. doi: 10.3389/fmicb.2020.593589 (PMC7746777; doi:10.3389/fmicb.2020.593589)
Supplement: Supplementary file 1 [file Data_Sheet_1.PDF]

Supplementary Material

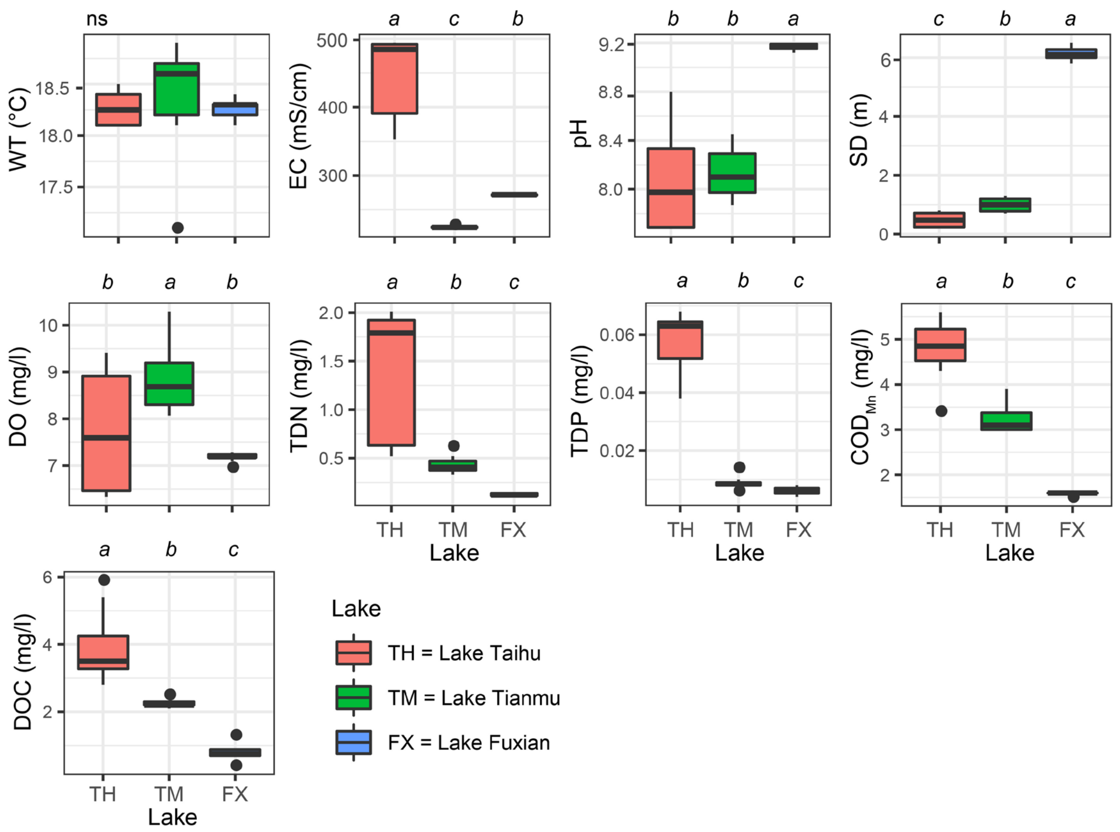

Supplementary Figure S1. Comparison of the nine environmental parameters among the three lakes. Horizontal bars in the box plots indicate median values. Lower and upper edges of the boxes represent the approximate 1st and 3rd quartiles, respectively. The lower and upper whiskers extend to data no more than 1.5 times the interquartile range from the upper edge and lower edge of the box, respectively. Dots above or below the boxes represent outliers. To examine differences among the three lakes, we performed the Kruskal-Wallis test. At the top of each panel, different lower-case letters indicate significant differences ($P < 0.05$) among different groups, while ns means non-significant ($P > 0.05$). WT, water temperature; EC, electrical conductivity; SD, Secchi disk transparency; DO, dissolved oxygen; TDN, total dissolved nitrogen; TDP, total dissolved phosphorus; COD$_{Mn}$, chemical oxygen demand; DOC, dissolved organic carbon.

Supplementary Table S1. Analysis of similarity (ANOSIM) of bacterial community structure between 2 habitats (PA, particle-attached; FL, free-living), and among different particle collection methods in Lake Taihu, Lake Tianmu and Lake Fuxian.

Comparison	Sample statistic *R*		
	Lake Taihu	Lake Tianmu	Lake Fuxian
Habitats			
PA vs FL	0.239*	0.656***	0.957***
Collection methods/Treatments			
Global ANOSIM	0.692***	0.994***	0.868***
>20 μm vs >10 μm	0.999*	0.999*	0.865*
>20 μm vs >8.0 μm	0.999*	0.999*	0.625*
>20 μm vs >5.0 μm	0.999*	0.999*	0.999*
>20 μm vs >3.0 μm	0.999*	0.999*	0.999*
>20 μm vs Precipitate	0.999*	0.999*	0.999*
>10 μm vs >8.0 μm	0.115	0.999*	0.042
>10 μm vs >5.0 μm	0.135	0.999*	0.896*
>10 μm vs >3.0 μm	0.001	0.999*	0.999*
>10 μm vs Precipitate	0.999*	0.999*	0.999*
>8.0 μm vs >5.0 μm	0.073	0.979*	0.823*
>8.0 μm vs >3.0 μm	0.021	0.999*	0.999*
>8.0 μm vs Precipitate	0.999*	0.999*	0.999*
>5.0 μm vs >3.0 μm	0.292	0.999*	0.917*
>5.0 μm vs Precipitate	0.999*	0.999*	0.999*
>3.0 μm vs Precipitate	0.999*	0.999*	0.999*

* $P < 0.05$; *** $P < 0.001$

Supplementary Table S2. Characteristics of filters of six different pore sizes (Merck Millipore Ltd.). All filters were hydrophilic, with a filter diameter (⌀) of 47 mm. More information can be found at http://www.merckmillipore.com/CN/en.

Catalogue Number	Pore size (μm)	Porosity (%)	Water Flow Rate (mL/min/cm²)*	Composition	Thickness (μm)
NY2004700	20	14	–	Nylon	–
TCTP04700	10	5–20	900	Polycarbonate	16
TETP04700	8.0	5–20	875	Polycarbonate	18
TMTP04700	5.0	5–20	550	Polycarbonate	20
TSTP04700	3.0	11.3	475	Polycarbonate	22
GTTP04700	0.2	13.8	16	Polycarbonate	25

* typical results at 10 psi.
